# Bivariate mixture models for the joint distribution of repeated serum ferritin and transferrin saturation measured 12 years apart in a cohort of healthy middle-aged Australians

**DOI:** 10.1371/journal.pone.0214196

**Published:** 2019-03-26

**Authors:** Christine E. McLaren, Wen-Pin Chen, Nadine A. Bertalli, Martin B. Delatycki, Graham G. Giles, Dallas R. English, John L. Hopper, Katrina J. Allen, Lyle C. Gurrin

**Affiliations:** 1 Department of Epidemiology, University of California, Irvine, California, United States of America; 2 Chao Family Comprehensive Cancer Center, Orange, California, United States of America; 3 Centre for Epidemiology and Biostatistics, School of Population and Global Health, The University of Melbourne, Melbourne, Australia; 4 Murdoch Childrens Research Institute, Melbourne, Australia; 5 Department of Paediatrics, The University of Melbourne, Royal Children’s Hospital, Melbourne, Australia; 6 Victorian Clinical Genetics Services, Melbourne, Australia; 7 Cancer Epidemiology and Intelligence Division, Cancer Council Victoria, Melbourne, Australia; 8 Department of Gastroenterology, Royal Children’s Hospital, Melbourne, Australia; Medizinische Universitat Innsbruck, AUSTRIA

## Abstract

Homozygosity for the p.C282Y substitution in the HFE protein encoded by the hemochromatosis gene on chromosome 6p (*HFE*) is a common genetic trait that increases susceptibility to iron overload. McLaren *et al*. used bivariate mixture modeling to analyze the joint population distribution of transferrin saturation (TS) and serum ferritin concentration (SF) measured for participants in the Hemochromatosis and Iron Overload Screening (HEIRS) Study. They identified four components (C1, C2, C3, and C4) with successively increasing means for TS and SF. They demonstrated that bivariate mixture modeling in TS and SF reflect the genetic locus of *HFE* and may isolate p.C282Y homozygotes from the general population. In the current study we used data from the another large cohort, the Australian HealthIron study of genetic and environmental modifiers of hereditary hemochromatosis, to validate the component analysis approach, to examine stability of component proportions over time and to determine if TS and SF values from an individual move between components at baseline and follow-up. Because sampling fractions from each p.C282Y / p.H63D genotype stratum are not equal, we used frequency weights based on the inverse of the probability of selection for invitation to participate. In the weighted female analytic cohorts, C4 captured most of C282Y homozygotes, and C2 was the largest component. We identified four components from the weighted male analytic cohort and C4 captured most of p.C282Y homozygotes. The bivariate mixture modeling approach suggested that the model is transferable from one white population to another, although estimated means within components may differ.

## Introduction

Hereditary Hemochromatosis (HH) is a genetic predisposition disease in which the body absorbs and accumulates excess iron from the diet. This excess iron is deposited in certain organs and, if left untreated, can cause tissue damage, the most serious example of which is cirrhosis of the liver, which can lead to liver failure and/or hepatocellular carcinoma and death. Approximately 90% of people with HH are homozygous for a polymorphism in the *HFE* gene (rs1800562) that causes substitution of the amino acid tyrosine for cysteine in the HFE protein at position 282 (p.Cys282Tyr; p.C282Y) [1]. The primary biochemical measures of iron status are transferrin saturation (TS), a measure of iron absorption, and serum ferritin (SF), a measure of iron accumulation.

McLaren *et al*. used bivariate mixture modeling to analyze the joint population distribution of transferrin saturation (TS) and serum ferritin concentration (SF) measured for participants in the Hemochromatosis and Iron Overload Screening (HEIRS) Study [2]. They identified four components (C1, C2, C3, and C4) with successively age-adjusted increasing means for TS and SF from data contributed by more than 100,000 participants of multiple ethnicities. They demonstrated that the bivariate mixture modeling in TS and SF reflect the genetic locus of *HFE* and has the potential to isolate p.C282Y homozygotes from the general population [2–4].

In the current study we used data from the another large cohort, the Australian HealthIron study of genetic and environmental modifiers of hereditary hemochromatosis (HH), to validate the component analysis approach, to examine stability of component proportions over time and to determine if TS and SF values from an individual move from one component at baseline to another at follow-up. We note that most new findings require replication before the original report can be considered to have been confirmed. Demonstrating replication is our intent.

## Methods

### Sources of data

Between 1990 and 1994, 41,514 people were recruited to participate in the Melbourne Collaborative Cohort Study (MCCS) [5]. Baseline blood samples from 31,192 participants of northern European descent (the remainder were from southern European countries such as Greece, Italy, and Malta, populations in which the p.C282Y polymorphism is rare) were genotyped for the p.C282Y and p.H63D polymorphisms in the *HFE* gene. An *HFE*-stratified random sample of these participants (n = 1,438) were selected for invitation to participate in the HealthIron study. Of these, 1,052 participated in interviews and had a clinical examination between 2004 and 2006.

The study originally named “*Genetics modifiers of haemochromatosis phenotype and environmental modifiers of clinical expression of haemochromatosis*” was approved by the Human Research Ethics Committee (HREC) of the Cancer Council Victoria (CCV), Australia as Project No. HREC_0105. The CCV’s HREC is a fully-constituted human research ethics committee consistent with the requirements of the Australian National Health and Medical Research Council’s “National Statement on Ethical Conduct in Human Research 2007 (Updated 2018)”.

The HealthIron participants are an *HFE*-genotype stratified random sample, not a screening cohort, so the sampling fractions from each p.C282Y/p.H63D genotype stratum are not equal. To reflect the *HFE* population proportions, we use frequency weights based on the inverse of the probability of selection for invitation to participate. These weights are 1 for p.C282Y homozygotes (all identified p.C282Y homozygotes were invited to participate), 3 for p.C282Y/p.H63D compound heterozygotes, 10 for p.C282Y simple heterozygotes and 50 for those with no p.C282Y and no p.H63D mutation. Participants who were previously treated for hemochromatosis, missing TS or SF values, missing age, and unknown genotype were excluded.

A data dictionary with variable names and a data spreadsheet are provided in a supporting information file that gives *HFE*-genotypes, demographic variables, and iron indices for HealthIron participants.

### Adjusted transferrin saturation and serum ferritin concentration

A restricted cubic spline analysis was used to reflect the non-linear relationships between TS and age [6, 7]. TS values were then adjusted for the age terms using separate multiple linear regression analyses by sex [6]. For each individual, the value of the regression residual was calculated and an adjusted transferrin saturation value was computed as the sum of the regression residual and a constant. The constant was calculated as the predicted TS at the median ages, which were 53.8 years at baseline and 65.5 years at follow-up for men, 53.2 years at baseline and 64.1 years at follow-up for women.

The distributions of serum ferritin concentration values were markedly skewed. The natural logarithm transformation was applied to induce the normality of the distributions. Following the same strategy stated above, the adjusted SF values were calculated for each individual at baseline and follow-up separately.

### Statistical mixture modeling of the bivariate distribution of TS and SF

A complete description of the use of mixture model methodology used to analze the bivariate distribution of TS and SF is provided in McLaren *et al*.[2]. In the current study we applied this bivariate mixture modeling approach separately at baseline and follow-up stratified by sex. The EMMIX program, which implements the EM algorithm, was utilized to fit models and to assess the number of normal components [8, 9]. The significance of the likelihood ratio test, the AIC and BIC statistics, and the estimate of overall correct allocation rate were used to determine the number of normal components to best fit the data [10–12]. The proportions and the means and variances for adjusted TS and adjusted SF within components of the bivariate distribution were computed. Allocation of each individual to a normal component was obtained at baseline and follow-up based on the highest posterior probability of component membership. A contingency table was constructed to display the shifting in the allocation from baseline to follow-up for individuals by *HFE* genotype, and Bowker’s test of symmetry was performed [13]. Scatter plots of adjusted TS and SF with 95% confidence ellipses based on the 4-component mixture models were constructed.

## Results

The final unweighted analytic sample consisted of TS and SF concentration values from 926 whites (426 men, 500 women) at baseline and in follow−up data from 771 whites (341 men, 430 women) (See Table 1). The final weighted sample included 23328 whites (10261 men, 13067 women) at baseline and in follow−up data from 21,045 whites (9,215 men, 11,830 women). (See Table 1). Table 2 displays the participants’ characteristics in each analytic cohort. Table 2 shows that in the weighted sample, male C282Y homozygotes comprised at follow-up 43% of the initial population (26/60), female C282Y homozygotes comprised at follow-up 68% of the initial population (49/72), and the other genotypes retained 43% of males (9189/10,201) and 68% of females (11,781/12,995). This may indicate that more male C292Y homozygotes were lost for follow-up after initial evaluation and could eventually develop a more severe phenotype.

**Table 1:** Exclusions for data from participants in the HealthIron study.

	Unweighted				Weighted			
Exclusion	Women Baseline	Women Follow-up	Men Baseline	Men Follow-up	Women Baseline	Women Follow-up	Men Baseline	Men Follow-up
Total Sample	783	783	655	655	16484	16484	13363	13363
Excluded for								
Not in Baseline Analysis Cohort	-	283	-	229	-	3417	-	3102
Missing values for TS	255	57	205	60	3331	1222	2931	919
Missing values for SF	2	1	3	1	60	3	150	50
Undetermined *HFE* genotype	26	0	18	0	26	0	18	0
Reported previous treated for iron overload	0	12	3	23	0	12	3	27
Age less than 25 or unknown	0	0	0	1	0	0	0	50
Final Analytic Sample	500	430	426	341	13067	11830	10261	9215

**Table 2:** Participants’ characteristics in the unweighted and weighted analytic samples.

	Unweighted				Weighted			
	Women Baseline	Women Follow-up	Men Baseline	Men Follow-up	Women Baseline	Women Follow-up	Men Baseline	Men Follow-up
Number of participants	500	430	426	341	13067	11830	10261	9215
Age-yr, mean (sd)	53.8 (9.1)	65.2 (9.3)	54.3 (9.1)	65.9 (9.1)	53.9 (9.3)	65.5 (9.5)	53.9 (9.2)	65.7 (9.2)
Serum Ferritin (ug/L), median range	83.5 (2.0–2781)	112.5 (3.0–1260)	234.0 (8.0–7134)	187.0 (4.0–1613)	77.0 (2.0–2781)	97.0 (3.0–1260)	186.0 (8.0–7134)	162.0 (4.0–1613)
Transferrin Saturation (%), mean (sd)	32.3 (16.1)	32.1 (14.9)	39.8 (19.1)	35.9 (16.9)	26.3 (9.6)	25.9 (9.6)	31.7 (11.0)	30.7 (11.3)
Clinical assessment at baseline								
Self-report diabetes, %	1 (5/500)	-	3 (12/426)	-	1 (163/13067)	-	3 (328/10261)	-
Self-report angina, %	2 (9/500)	-	6 (24/426)	-	1 (178/13067)	-	6 (569/10261)	-
Self-report heart attack or myocardial infarction, %	1 (6/500)	-	5 (21/426)	-	1 (108/13067)	-	5 (491/10261)	-
Clinical assessment at follow-up								
Self-report liver disease, %	-	8 (34/419)	-	3 (11/335)	-	7 (787/11561)	-	4 (369/9042)
Self-report fatigue, %	-	18 (74/421)	-	10 (32/334)	-	18 (2081/11614)	-	11 (1029/9081)
Abnormal 2^nd^ and 3^rd^ MCP joints on either hand, %	-	12 (41/354)	-	16 (48/295)	-	11 (1048/9785)	-	17 (1325/8027)
Liver enlargement, %	-	1 (5/342)	-	4 (11/289)	-	2 (154/9359)	-	4 (287/7901)
Genotype								
HO (C282Y homozygote)	72	49	60	26	72	49	60	26
HEHE (C282Y/H63D compound heterozygote)	75	67	77	63	225	201	231	189
HEN (C282Y heterozygote)	122	103	112	90	1220	1030	1120	900
NHE (H63D heterozygote)	63	55	38	35	3150	2750	1900	1750
NHO (H63D homozygote)	8	8	6	6	400	400	300	300
NN (HFE wildtype)	160	148	133	121	8000	7400	6650	6050

Note: ‘-‘ represents ‘not applicable’.

**Fig 1:**
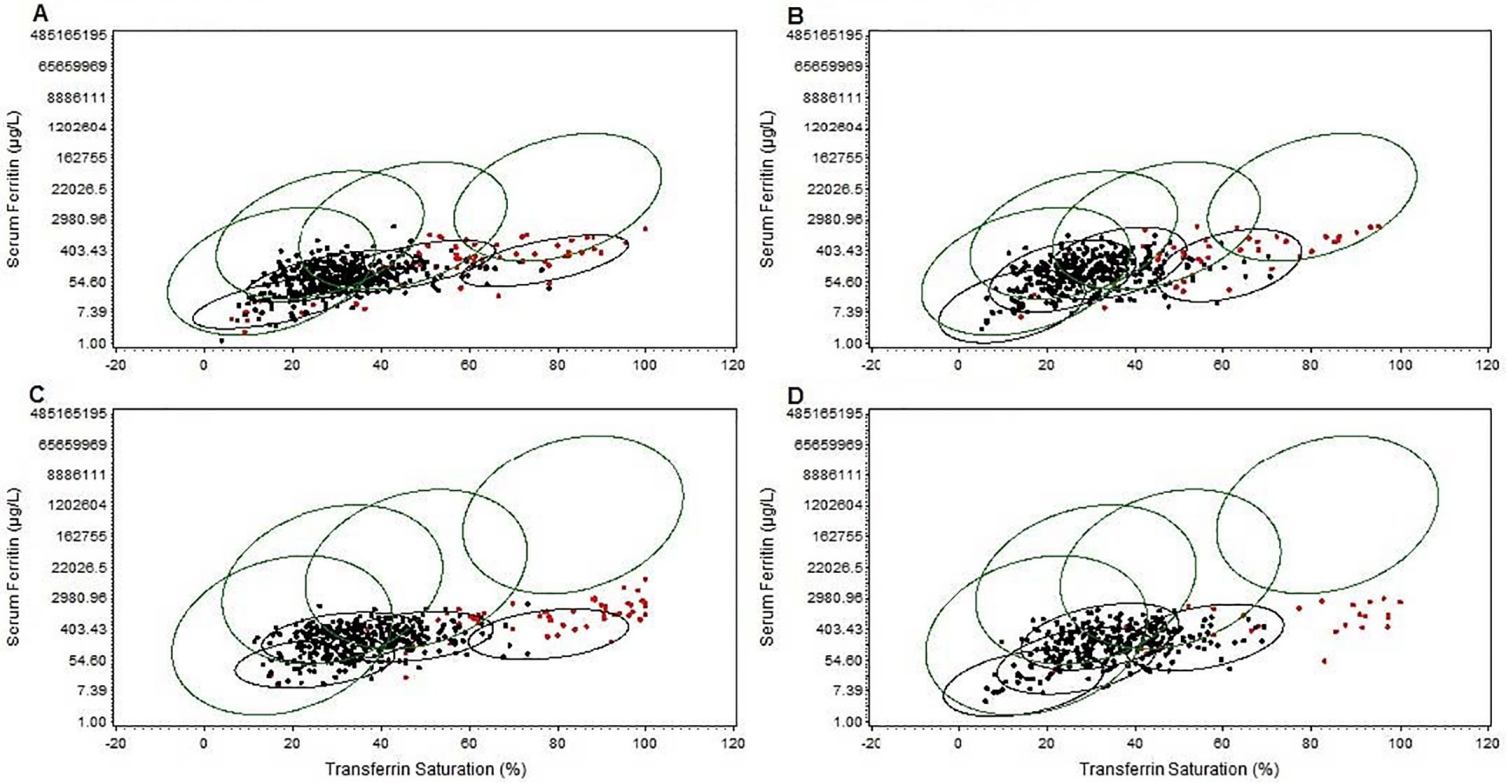
Scatter plots of adjusted TS and SF with indication of the C282Y homozygote cases as red dots from HealthIron study. The 95% confidence ellipses in black and in green were based on the 4-component models from HealthIron and HEIRS data, respectively. The weighted analytic cohorts: A. Female at baseline, B. Female at follow-up, C. Male at baseline and D. Male at follow-up.

Table 3 displays the results of an analysis with four components for both the weighted female and weighted male analytic cohorts, respectively. Although there was some statistical evidence to support the five-component model, we chose to work with the four-component model to aid the comparison of our results with those of previously published research [2]. The largest component, C2, had normal mean TS (28% at baseline, 23% at follow-up) and SF (77 μg/L at baseline, 78 μg/L at the follow-up), with component proportion of 0.82 at baseline and 0.63 at follow-up respectively. In the weighted male cohorts, we determined 4 components at baseline and at the follow-up separately. At baseline, the largest component, C2, had normal mean TS value of 31% and a normal mean SF of 243 μg/L. However, at the follow-up, the largest component is C3 with a normal mean TS value of 32% and a normal mean SF of 250 μg/L. Fig 1 displays the scatter plots of adjusted TS and SF values with the indication of p.C282Y homozygotes in red dots from the weighted HealthIron data. The 95% confidence ellipses in black and green color were added based on the results of mixture models from HealthIron and HEIRS data, respectively.

**Table 3:** The estimated proportions and the means and variances for adjusted TS and adjusted SF within components of the bivariate distribution in the weighted analytic cohort by sex.

Cohort	Mixture component	Estimate of mixing proportion	Estimated adjusted mean TS (%)	Estimated adjusted TS variance	Estimated adjusted mean SF (ug/L)	Estimated adjusted mean SF (ug/L) in original scale^1^	Estimated adjusted SF variance
Women at Baseline N = 13,067	C4	0.003	77.060	43.834	5.344	209.369	0.592
	C3	0.057	46.735	43.834	4.993	147.304	0.592
	C2	0.821	28.351	43.834	4.338	76.524	0.592
	C1	0.118	16.299	43.834	2.638	13.984	0.592
Women at Follow-up N = 11,830	C4	0.009	61.187	36.233	5.049	155.804	0.724
	C3	0.288	35.218	36.233	5.185	178.573	0.724
	C2	0.627	23.479	36.233	4.351	77.587	0.724
	C1	0.076	12.083	36.233	2.384	10.853	0.724
Men at Baseline N = 10,261	C4	0.010	77.955	56.098	5.671	290.441	0.477
	C3	0.160	47.121	56.098	5.513	247.819	0.477
	C2	0.686	31.046	56.098	5.454	233.668	0.477
	C1	0.145	24.993	56.098	3.831	46.104	0.477
Men at Follow-up N = 9,215	C4	0.068	55.619	62.295	5.472	237.959	0.547
	C3	0.638	32.073	62.295	5.522	250.135	0.547
	C2	0.234	26.722	62.295	3.953	52.097	0.547
	C1	0.060	14.619	62.295	2.503	12.219	0.547

^1^Estimated adjusted SF in original scale = exp^(Ln(SF))^

Female p.C282Y homozygotes showed evidence that component transition probabilities shifted significantly over time; 29% had TS and SF values that were in the same bivariate TS/SF component at baseline and follow−up (Bowker’s test of symmetry, p = 0.03). For the other genotypes, the percentages of females who had TS and SF values that were in the same bivariate component at baseline and follow−up were 40% for p.C282Y/p.H63D compound heterozygote, 47% for p.C282Y heterozygotes, 49% for p.H63D heterozygotes, 75% for p.H63D homozygotes and 68% for *HFE* wildtype. In the male cohort, the percentages of participants whose TS and SF values were in the same bivariate component at baseline and follow−up varied from 33% to 77% by genotype (Bowker’s test of symmetry, all p-values < 0.0001, except for p.C282Y homozygotes where p = 0.33) (see Tables 4 and 5).

**Table 4:** The contingency table of baseline versus follow-up group membership among females (N = 11830).

**Baseline component**	**Follow-up Component**				
	**1**	**2**	**3**	**4**	**Total**
1	150	736	283	6	1175
2	490	6823	2724	35	10072
3	60	165	295	45	565
4	0	2	9	7	18
Total	700	7726	3311	93	11830

**Table 5:** The contingency table of baseline versus follow-up group membership among males (N = 9215).

**Baseline component**	**Follow-up Component**				
	**1**	**2**	**3**	**4**	**Total**
1	273	399	380	101	1153
2	236	1416	4964	244	6860
3	50	146	775	161	1132
4	0	51	1	18	70
Total	559	2012	6120	524	9215

## Discussion

We applied bivariate mixture modeling to analyze joint population distributions of TS and SF measured in the HealthIron Study. We identified four components with successively age-adjusted increasing means for TS and SF at baseline and follow-up data by sex, separately. In the weighted female analytic cohorts, C4 captured most of p.C282Y homozygotes, and C2 was the largest component. In our study at baseline and follow-up, the means of adjusted TS and SF within components were higher than the means from HEIRS data. The estimated variances from the HealthIron data were much smaller than the estimated variances from HEIRS. In addition, we identified 4 components from the weighted male analytic cohort and C4 captured most of the p.C282Y homozygotes. Therefore, the bivariate mixture modeling approach suggested that the model is transferable from one white population to another, although estimated means within components may differ. The longitudinal aspect of this study is unique and illustrates that, with the exception of female p.C282Y homozygotes, the components of the mixture distributions are largely stable over time.

Strengths of this study include the prospective nature of recruitment for both the HEIRS and HealthIron studies and the comprehensive phenotyping through multiple sources: self-completed questionnaires, standardized biochemical analysis of all blood and tissue samples, and comprehensive clinical examination of participants by study physicians. Weaknesses include the different circumstances, motivations and time periods under which the two cohorts were recruited, and the different sampling fractions for each *HFE*-genotype group in the HealthIron study. This required the use of frequency weights to implement the mixture model analysis for which only approximate measures of precision of estimation are available.

In conclusion, our study provides another demonstration of the usefulness of mixture modeling in determining the presence and estimating the frequency of subpopulations in an overall population distribution. A unique aspect of our investigation was the access to longitudinal data enabling determination of subpopulation components at baseline and follow-up and assessment of a shift of component probabilities over time. A significant shift between baseline and follow-up was found for female p.C282Y homozygotes but not for male p.C282Y homozygotes.

## Supporting information

S1 File A data dictionary with variable names and a data spreadsheet are provided in a supporting information file. The data spreadsheet gives *HFE*-genotypes, demographic variables, and iron indices for HealthIron participants.
(XLS)
